# Metabolic Profile of Einkorn, Spelt, Emmer Ancient Wheat Species Sourdough Fermented with Strain of *Lactiplantibacillus plantarum* ATCC 8014

**DOI:** 10.3390/foods12051096

**Published:** 2023-03-04

**Authors:** Larisa Rebeca Șerban, Adriana Păucean, Maria Simona Chiș, Carmen Rodica Pop, Simona Maria Man, Andreea Pușcaș, Floricuța Ranga, Sonia Ancuța Socaci, Ersilia Alexa, Adina Berbecea, Cristina Anamaria Semeniuc, Vlad Mureșan

**Affiliations:** 1 Department of Food Engineering, Faculty of Food Sciences and Technology, University of Agricultural Sciences and Veterinary Medicine of Cluj-Napoca, 3-5 Mănăștur Street, 400372 Cluj-Napoca, Romania; 2 Department of Food Science, Faculty of Food Science and Technology, University of Agricultural Sciences and Veterinary Medicine of Cluj-Napoca, 3-5, Mănăștur Street, 400372 Cluj-Napoca, Romania; 3 Faculty of Food Science and Technology, Institute of Life Sciences, University of Agricultural Sciences and Veterinary Medicine of Cluj-Napoca, 3-5 Mănăștur Street, 400372 Cluj-Napoca, Romania; 4 Department of Food Control, Faculty of Agro-Food Technologies, University of Life Sciences “King Michael I of Romania”, 119 Aradului Avenue, 300641 Timişoara, Romania; 5 Department of Soil Sciences, Faculty of Agriculture, University of Life Sciences “King Michael I of Romania”, 119 Aradului Avenue, 300641 Timişoara, Romania

**Keywords:** ancient, einkorn, spelt, emmer, sourdough, *Lactiplantibacillus plantarum*, metabolic

## Abstract

The continuous development of bakery products as well as the increased demands from consumers transform ancient grains into alternatives with high nutritional potential for modern wheat species. The present study, therefore, follows the changes that occur in the sourdough obtained from these vegetable matrices fermented by *Lactiplantibacillus plantarum* ATCC 8014 during a 24 h. period. The samples were analyzed in terms of cell growth dynamics, carbohydrate content, crude cellulose, minerals, organic acids, volatile compounds, and rheological properties. The results revealed significant microbial growth in all samples, with an average value of 9 log cfu/g but also a high accumulation of organic acids with the increase in the fermentation period. Lactic acid content ranged from 2.89 to 6.65 mg/g, while acetic acid recorded values between 0.51 and 1.1 mg/g. Regarding the content of simple sugars, maltose was converted into glucose, and fructose was used as an electron acceptor or carbon source. Cellulose content decreased as a result of the solubilization of soluble fibers into insoluble fibers under enzymatic action, with percentages of 3.8 to 9.5%. All sourdough samples had a high content of minerals; the highest of which—Ca (246 mg/kg), Zn (36 mg/kg), Mn (46 mg/kg), and Fe (19 mg/kg)—were recorded in the einkorn sourdough.

## 1. Introduction

One of the earliest types of natural starters is sourdough, which is typically used as an alternative to baker’s yeast for preparing leavened baked items. As endogenous lactic acid bacteria (LAB) and/or yeasts are naturally present in the raw materials, this was actually the original method of manufacturing bread through natural leavening before commercial yeast was utilized for leavening [1,2,3]. At present, starter cultures of lactic acid bacteria (LAB) are mostly used for the fermentation of bakery products on a large scale because they control the fermentation process and the quality of the final product [4,5]. Other advantages of starter cultures are decreasing fermentation times, reducing energy costs, improving the sensory qualities of the products, and minimizing their spoilage risks [6].

The sourdough-making process involves a mixture of flour, water, and lactic acid bacteria fermented at a constant temperature for a predefined period [7,8,9]. Nowadays, sourdough technology has gained popularity among bakers due to its ability to enhance bread quality [10,11,12,13,14]. Thus, organic acids produced by LAB metabolism increase the shelf life of the bread, prevent its fungal and bacterial alteration, restrict the activity of endogenous amylase, and have a great effect on the dough’s capacity to bind water and hold gas [15,16,17]. At the same time, sourdough plays a significant role in a variety of other aspects, such as improving the rheological properties of bread, increasing the bioavailability of minerals, protein digestibility and soluble fibers, contributing to the formation of flavor compounds, reducing the glycemic index, lowering the levels of phytate and trypsin inhibitors, and others [18,19,20,21].

Lactic acid bacteria (LAB) are some of the most commonly used microorganisms in the food industry. Due to their diversity and variability, LAB offers an endless source of perspectives for technological processes [22]. *Lactiplantibacillus plantarum* (*Lb. plantarum*) is a facultative heterofermentative bacteria extensively used in the bakery, which is recognized and appreciated for the structure and acidification it provides to the cereal grains used for fermentation [23,24]. *Lb. plantarum* is a versatile bacterial strain that can adapt to different substrates [25]. It was successfully used in sourdough together with a wide variety of cereals, such as Kamut^®^ wheat (*Lb. plantarum* M4), wheat flour (*Lb. plantarum* ATCC 14917, *Lb. plantarum* ATCC 8014), emmer wheat (*Lb. plantarum* 6E, *Lb. plantarum* 10E), spelt wheat (*Lb. plantarum* ATCC 31S), rye, oats, and barley (*Lb. plantarum* LUHS135) [26,27,28,29,30]. Nevertheless, due to the interaction between the raw matrix (flour) and microbial activity, a deep understanding of the metabolic profile of different types of dough is still needed.

Due to their unique chemical composition, ancient grains like einkorn, emmer, and spelt have retracted the interest of consumers; also, farmers are interested and enticed by their low cultivation and maintenance requirements [20,31,32,33,34,35,36,37,38]. Between the compounds of interest, β-glucans, essential amino acids, phenols, proteins, and minerals are considered highly important and studied. Moreover, compared to common wheat and durum wheat, einkorn stands out for its higher content of total phenolic compounds (2.06–8.11 µmol GAE/g), lipids 2.4–3.2% with a high content of mono-unsaturated fatty acids (26.85%), poly-unsaturated fatty acids (56.55%), ferulic acid (148.67–764.04 µg/g), p-coumaric acid (5.06–54.09 µg/g), and certain minerals, such as zinc (5.4 mg/100 g) [39,40]. Being richer in lutein (90% of total carotenoids) than modern wheat, the emmer variety’s remarkable nutritional value is provided by its high level of antioxidant compounds, and dietary fibers, (11.5–15.5%) such as cellulose, arabinoxylans, and β-glucans, which are the major components of the grain’s cell wall [31,41]. Regarding spelt, it is recognized for its high percentage of proteins (15.17% emmer wheat vs. 11.58% common wheat), and vitamins such as niacin (5.5 mg/100 g), which is found in a higher quantity in this cereal than in einkorn or wheat (2.5 mg/100 g) [42,43].

All these qualities offer ancient wheat species flours a uniqueness that can lead to the development of innovative, healthy, and functional products. Several studies have revealed the health-beneficial nature of these grains, such as their involvement in the prevention and alleviation of some diseases such as diabetes type 2, cancer, obesity, coronary heart disease, ischemic stroke, osteoporosis, and others [44,45,46]. Since the technological properties of these ancient species are inferior to those of modern wheat species and standard processing technologies cannot be applied, researchers have begun to adopt and develop new methods, strategies, and protocols to obtain high-quality bakery products [47,48].

Metabolomics is the approach that can provide a more specific perspective through observing the evolution of the sourdough’s profile during the fermentation process. As a general definition, metabolic represents a complex research field aiming to study the biochemical processes that involve small metabolites. These analyses are composed of several stages, including sample preparation, data acquisition, data processing, analysis, and interpretation of the results [49]. The most importance are separation and detection, while a few of the most frequently used techniques of separation are high-performance liquid chromatography (HPLC), gas chromatography (GC), mass spectrometry (MS), frequently ultraviolet (UV), nuclear magnetic resonance (NMR), and near -infrared (NIR) spectrometry [50]. Additionally, another important part of metabolomics is related to statistical models on metabolite profiles, which are designed to anticipate variables that are difficult to determine in other ways [49]. Depending on the purpose of the metabolic approach, it can have three roles, including informative, descriptive, and predictive [49]. In the bakery sector, metabolomics is used mainly to determine the effect that the type of flour used and fermentation have on the formation of volatile compounds, but also to quantify carbohydrates, amino acids, organic acids, and other specific compounds [51,52].

The literature review on the topic of ancient wheat species revealed few studies that reported on the metabolic profile of sourdough obtained by fermenting these flours with *Lactiplantibacillus plantarum* strains. Moreover, the existing studies report mainly on Durum wheat, spelt, and KAMUT^®^ khorasan wheat [53,54,55,56,57] and a wider image of other ancient wheat species like emmer and einkorn that was not reported. In addition, the volatile derivative content of sourdough needs to be deeply investigated since these compounds play a highly important role in the bread’s sensory characteristics. In this view, the present research study aimed to use a metabolomic approach to assess the adaptability of *Lactiplantibacillus plantarum* ATCC 8014 in the sourdough obtained from ancient wheat flours (einkorn, spelt, and emmer) in order to be used in breadmaking. This approach could give a more complete image to the performance of these wheat species for sourdough production by providing a comparison between their metabolic profiles and common wheat, which is mainly used in breadmaking. This will provide the possibility to monitor the traceability of the bioactive compounds from the raw flour to bread via sourdough technology and to obtain bakery products fortified with these biocompounds with good sensorial features due to the specific aroma compounds.

## 2. Materials and Methods

### 2.1. Materials

Whole meal flours (einkorn, spelt, emmer, and common wheat) were purchased from specialized stores in Romania. *Lactiplantibacillus plantarum* ATTC 8014 was acquired from Microbiologics (Minnesota, USA), and all reagents and chemicals used for analysis came from Sigma Aldrich (Taufkirchen, Germany) and Chempur (Piekary Śląskie, Poland), and were of analytical grade. The equipment used included the following: laboratory glassware, analytical balance, technical balance, pH meter (GroLine H1285-7, Woonsocket, Rhode Island, USA), furnace (Nabertherm B150, Lilienthal, Germany), centrifuge (Eppendorf AG 5804, Hamburg, Germany), vortex (Heidolph Reax Top vortex), Shimadzu UV-1900 (Shimadzu Scientific Instruments, Kyoto, Japan), optical microscope (Zeiss 40X, Primo Star, Germany), colony counter (Colony Star 8500, Funke Gerber, Berlin, Germany), Agilent 1200 HPLC System (Agilent Technologies, Santa Clara, CA, USA), FOSS 2010 (Fibertec 2010, Hillerød, Denmark), Varian 220 FAA equipment (Germany), Gas Chromatograph Mass Spectrometer QP 2010 (Shimadzu Scientific Instruments, Kyoto, Japan), Anton Paar MCR 302 rheometer (Anton Paar, Graz, Austria).

### 2.2. Sourdough Formulation, Lactiplantibacillusplantarum ATCC 8014 Activation, and Cell Count Determination

Shortly, the inoculum was obtained from freeze-dried cells suspended in10 mL Man Rogosa Sharpe (MRS) broth, incubated under aerobic conditions at a temperature of 37 °C for 48 h, and then sub-cultured into 95 mL MRS and incubated in the same conditions. Afterward, the biomass was centrifuged at 2300× *g* (Eppendorf R 5804 centrifuge, Hamburg, Germany) for 10 min, at a temperature of 4 °C, washed three times with sterile water, and inoculated in the prepared matrix in order to achieve an initial cell count of 10^8^ cfu/mL. The microbial optical density of the inoculum was determined using the spectrophotometer Shimadzu UV-1900 (Shimadzu Scientific Instruments, Kyoto, Japan), and absorbance was read at a wavelength of 600 nm [58].

Sourdough samples were obtained by mixing flour with distilled water at a dough yield of (DY = 1:0.8), while *Lb. plantarum* strain was added at a level of 10^8^ cfu/mL to the mixture. Samples were taken at 0, 12, and 24 h of fermentation at 35 °C and analyzed for microbial cell growth dynamics. To determine the increase in cfu/g, decimal dilutions were performed, and 1 mL of each sourdough was mixed with 9 mL of saline solution. In a Petri dish with MRS agar, 1 mL of the sample was added and incubated for 48 h at 37 °C [58]. The microbiological analysis was performed in three replicates (*n* = 3).

At sampling times, 5 mL of each sample was taken out and combined with 45 mL of sterile sodium chloride (0.85% *w*/*v*). One milliliter of this solution was used for serial dilutions and plating on MRS agar under the following incubation: 37 °C, 48 h. The final stage involved the analysis of Petri plates with colonies under an optical microscope (Zeiss 40X, Primo Star, Germany) in order to identify microorganisms, while for counting, a colony counter (Colony Star 8500, Funke Gerber, Berlin, Germany) was used [24].

The formulations for the four types of sourdough (einkorn, spelt, emmer, and common wheat as a control sample) and their codifications are shown in Table 1.

### 2.3. Sourdough Acidification—Total Titratable Acidity (TTA) and pH Determination

The pH was determined using a pH meter (GroLine HI1285-7, Woonsocket, RI, USA) after it was initially calibrated with a standard solution.

The total titratable acidity (TTA) was determined by blending 10 g of sourdough with 90 mL of water, followed by the neutralization of the mixture obtained with NaOH 0.1 N until the pH dropped to 8.3. Finally, the total acidity is expressed as the volume (mL) of NaOH used in the titration [59,60].

### 2.4. Determination of Carbohydrates, Organic Acids, and Ethanol Content by HPLC-RID

The identification of carbohydrates (maltose, glucose, and fructose), organic acids (lactic, acetic, and citric), and ethanol was carried out with the help of the Agilent 1200 series HPLC system, which was equipped with quaternary pumps, a solvent degasser, and a manual injector coupled with a refractive index detector (RID). Agilent Technologies, CA, USA, provided the Polaris Hi-Plex H column, 300 × 7.7 mm, which was utilized to separate the compounds. The mobile phase H_2_SO_4_ 5 mM was used at a flow rate of 0.6 mL/min, column temperature T = 80 °C, and RID temperature T = 35 °C; compounds were eluded for 25 min. For result interpretation, the OpenLab—ChemStation (Agilent Technologies, Santa Clara, CA, USA) system was used. In the end, obtained retention times were compared with standard times for glucose, fructose, maltose, citric acid, lactic, acetic acid, and ethanol (Sigma-Aldrich, Germany) to identify the compounds.

Briefly, 2 g of the sample and 4 mL of ultrapure water (UPW) is vortexed (Heidolph Reax Top vortex) for 1 min, sonicated for 30 min (Elmasonic E15H sonication bath), and centrifuged (Eppendorf AG 5804 centrifuge) at 7155× *g* for another 10 min. The resulting supernatant is filtered using a 0.45 µm nylon filter (CHROMAFIL Xtra PA-45/13), and 20 µL of this is injected into the Agilent 1200 HPLC system [58].

### 2.5. Determination of Crude Fiber Content (Crude Cellulose)

Crude cellulose was determined according to the standardized method (ISO 5498:1981) that was performed using the fiber analyzer FOSS 2010 (Fibertec 2010, Hillerød, Denmark). Briefly, 1 g of the sample was defatted by washing it three times with acetone. The defatted sample was boiled with sulfuric acid 12,5% for 30 min, washed with distilled water, and boiled with KOH 12,5% for 30 min. The resulting sample was calcined in an oven (BINDER GmbH, Tuttlingen, Germany) at 525 °C for 3 h, cooled and weighed. Crude cellulose was calculated as the ratio between the sample weight after calcination and the initial weight of the sample [61].

### 2.6. Determination of Micro and Macroelements by Atomic Absorption Spectrophotometry (AAS)

Macro and microelements contained in sourdough were identified using an atomic absorption spectrophotometry (ASS) (Varian 220 FAA Atomic Absorption Spectrometer, Varian Inc., Germany.

The samples (3 g) were analyzed with the Varian 220 FAA equipment after preliminary processing, which consisted of their calcination for 10 h at 500 ± 100 °C in a furnace (Nabertherm B150, Lilienthal, Germany). The resulting residue was then treated with 5 mL of HCl 6 mol/L and subsequently dissolved in an exact volume, 20 mL of HNO_3_ 0.1 mol/L. The values obtained at the end of the analysis are expressed as parts per million (ppm), each being the average of three independent determinations [62].

### 2.7. Determination of Volatile Compounds by ITEX/GC-MS Technique

The ITEX/GC-MS technique was used for the analysis of aroma compounds and assumed the use of the CombiPAL AOC-5000 autosampler. in which 1 g of each sample was inserted, sealed, and incubated for 20 min at 60 °C, under continuous stirring. At the end of the incubation, the volatile compounds accumulated in the headspace phase were adsorbed into a Tenax carbon fiber (ITEX-2TRAPTXTA, Tenax TA 80/100 mesh) and subsequently thermally desorbed in the gas chromatograph injector [63,64].

GCMS QP-2010 (Shimadzu Scientific Instruments, Kyoto, Japan) mass spectrometer performed the separation of aroma compounds on a ZB-5ms capillary column of 30 m × 0.25 mm i.d. × 0.25 µm (film thickness). The chromatographic column used the following temperature program: in the first phase, 35 °C was held for 5 min, followed by an increase to 110 °C with 4 °/min in the second phase, and an increase to 250 °C with 20 °/min for another 5 min in the third phase. Helium was used as a carrier gas, at a constant flow rate of 1 mL/min.; also, the temperature for the injector, ion source, and interface was chosen to be 250 °C. The mass spectrometry detector was operated in electron impact ionization mode over a scan range of 40–400 *m*/*z* [64,65].

The identification of volatile compounds was achieved by comparing the mass spectra of each chromatographic peak with the NIST27 and NIST147 libraries, considering only compounds that registered a degree of similarity of at least 85%.

### 2.8. Determination of Rheological Properties

The rheological measurements of the sourdough were realized with an Anton Paar MCR 302 rheometer (Anton Paar, Graz, Austria), using a parallel plate geometry (PP50) with a diameter of 50 mm. The method assumes placing 3 g of each sample on the lower plate of the device and lowering the upper plate to a plate distance set at a gap of 1 mm. The next steps consist of cleaning the sourdough surplus resulting from the pressing and adding silicone oil in order to avoid reducing the moisture of the sample through testing. The working temperature of the rheometer was set at 25 °C, and the storage modulus (G’) and the loss modulus (G″) were tested at an angular frequency of 0.628–628 rad/s^−1^, and the shear deformation was set at a value of 0.1% [66,67].

### 2.9. Statistical Analyses

The Duncan multiple comparison test (SPSS version 19 software version 19; IBM Corp., Armonk, NY, USA) was used to compare the obtained data. The analyses were performed in three independent assays, and small letters indicated the significant differences (*p* ˂ 0.05) between the 4 types of sourdough at the same moment.

Principal component analysis (PCA) was performed using the Unscrambler software (version 10.5.1; CAMO Software AS, Oslo, Norway), while the Hierarchical Cluster Analysis (HCA) and Heatmap Visualization were performed with MetaboAnalyst software (version 5.0; Xia Lab at McGill University, Quebec, QC, Canada).

## 3. Results and Discussion

### 3.1. Cell Viability in Sourdough Samples

**Figure 1:**
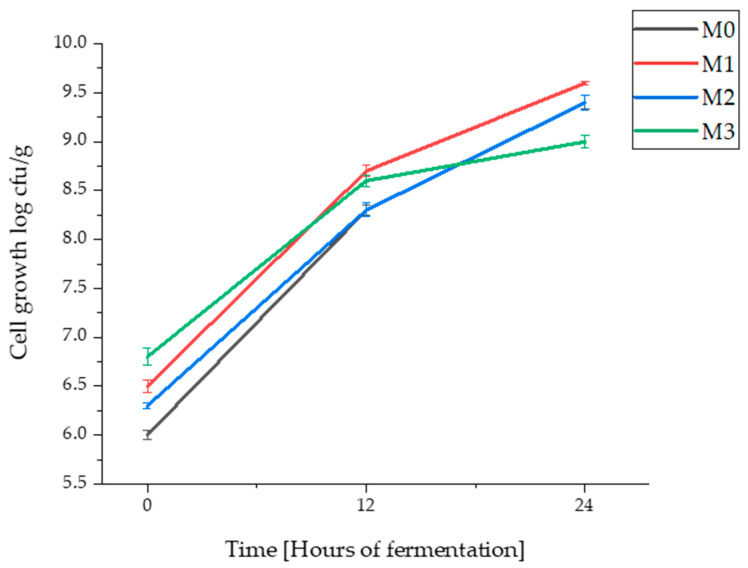
Cell growth in sourdough with wheat flour (M_0_), einkorn flour (M_1_), spelt flour (M_2_), and emmer flour (M_3_), fermented for 0, 12, and 24 h with *Lactiplantibacillus plantarum* ATCC 8014. Results are represented as mean values ± standard deviation (SD); *n* = 3.

In Figure 1, significant bacterial cell growth of *Lactiplantibacillus plantarum* ATCC 8014 can be observed in all four types of wheat flour sourdoughs (common and ancient wheat species), demonstrating the adaptability that *Lb. plantarum* ATCC 8014 has in these flours. The microbial growth dynamic in the control sample (M_0_ wheat flour sourdough) started at a value of 6.0 log cfu/g to when it registered after 24 h cell growth of 9.4 log cfu/g. The highest final concentrations, after 24 h of fermentation were recorded, for sample M_1_ (einkorn flour sourdough) at 9.6 log cfu/g, followed by M_2_ sample (spelt flour sourdough) reaching 9.4 log cfu/g after 24 h. The lowest growth, but still appreciable, was determined in the sample with the emmer flour (M_3_) which began at 6.8 log cfu/g and reached 9.0 log cfu/g at the end of the fermentation period. After 24 h of fermentation, significant differences (*p* < 0.05) between the microbial dynamics of the four types of flours were recorded.

Similar results were reported by Çakır et al. [68], who recorded that in einkorn sourdough fermented with different strains of *Lb. plantarum* (AAS3, FM02, 1838, GM1043), values between 9.26 log cfu/mL and 9.47 log cfu/mL (after 24 h fermentation). In another study, the reported values went above 9.0 log cfu/g (after 24 h fermentation) in the sourdough obtained from common wheat and *Lb. plantarum* M4 [26].

The most important aspect in terms of LAB growth and viability is the nutrients availability. It was stated that einkorn, spelt, and emmer contained high amounts of proteins, amino acids, vitamin E, vitamins of B-group, and minerals like calcium, magnesium, iron and zinc, which are strongly necessary for *Lactobacillus* ssp. growth [69]. The microorganisms consume firstly free amino acids, vitamins, and simple sugars, all compounds that are easily metabolized. After that, the growth of *Lb.plantarum* depended on its ability to breakdown the protein chain into the peptides and amino acid necessary to meet its nitrogen requirements [24]. As it was reported in our previous work, Șerban et al. [20], einkorn and spelt have the highest protein content compared to emmer and common wheat and this aspect could explain their different microbial growth during 24 h. Coda et al. [70] stated in their research that the proteolysis of spelt flour leads to essential amino acids (isoleucine, leucine, valine, and methionine), which on the one hand sustain bacterial metabolism and on the other hand contribute to the health benefits by supporting the production of bioactive peptides.

Regarding carbohydrates, *Lactobacillus* ssp. uses them as a carbon source to sustain the development of the microbial cells. The studied flours are rich in simple sugars, which are primarily used for microbial growth at the beginning of fermentation. Compared to common wheat flour (0.41 g/100 g), spelt (2.94 g/100 g) and einkorn (2.67 mg/100 g) contain a significantly higher amount of simple sugars [20,40]. These simple sugars initiated cell multiplication, giving a good start for the ancient wheat species, as it is sustained by the values obtained after 12 h of fermentation.

Vitamins from B -group such as thiamine (einkorn—1.118 µg/g, spelt—3.46 µg/g, emmer—0.952 µg/g, wheat—0.964 µg/g), riboflavin (einkorn—1.118 µg/g, spelt—1.64 µg/g, emmer—0.952 µg/g, wheat—0.964 µg/g), and niacin (einkorn—55 µg/g, spelt—66 µg/g, emmer—85.11 µg/g, wheat—47.66 µg/g) supports the bacterial growth in these flours [20,71,72].

Thus, the chemical composition of einkorn, spelt, and emmer could sustain the cell dynamics of *Lb. plantarum* during 24 h of fermentation and give an advantage in cell development compared to common wheat. However, the differences recorded on the final cell count might be due to the variations in flour quality based on provenance, environment, production practices, and storage conditions [73].

According to Clément et al. [74], flour ash content also has an important role in microbial growth, in wheat sourdough with a high mineral content recording a cell growth from 9.9 × 10^7^ to 6 × 10^8^ cfu/g in 48 h of fermentation. It is important to mention that this content is influenced by two main factors, namely the flour extraction rate and the milling process [74]. In the present study, all flour samples were whole meal flours with a high content of minerals, which also sustained the bacterial growth.

Other factors that can influence microbial growth are those related to water activity; the required values ranging between 0.90 and 0.96 for *Lactobacillus* species [75]. Thus, of great relevance is the availability of water in a sourdough starter. This factor refers to dough yield (DY = [flour weight + water weight] × 100/flour weight) and hydration (the percent of water to flour) [9]. The importance of hydration is demonstrated by the fact that water diffuses proteolytic enzymes and nutrients, and influences the composition and activity of the bacteria from the starter [9]. According to Di Cagno et al. [76] and Minervini et al. [77], who studied sourdoughs obtained from durum wheat (*Triticum durum*), *Lb. plantarum* prefers and dominates in firm ones, which present a dough yield (DY) between 150 and 200. In the case of the present study, DY = 180 was used to sustain the microbial cell dynamics.

In this regard, we can assume that ancient wheat species, namely einkorn, spelt, emmer, are a good matrix for the growth of *Lb. plantarum* ATCC 8014.

### 3.2. pH and TTA Values

**Figure 2:**
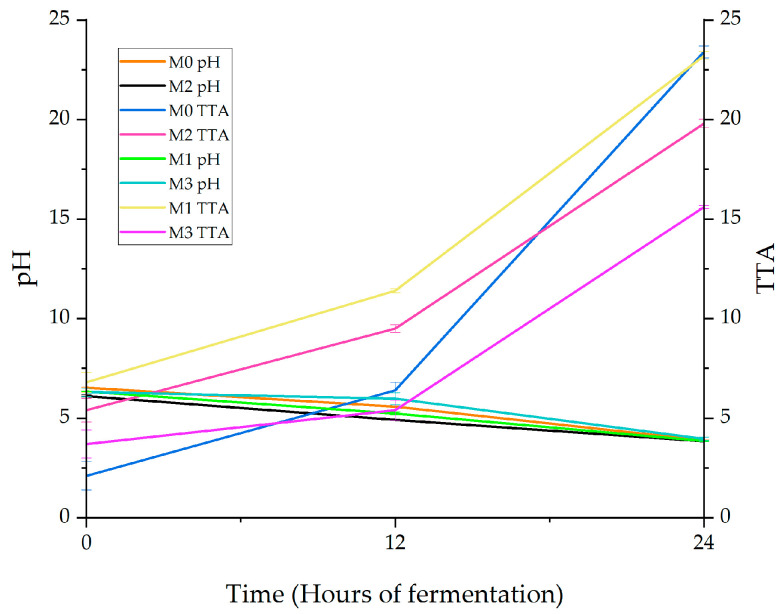
pH and TTA in sourdough with wheat flour (M0), einkorn flour (M1), spelt flour (M2), and emmer flour (M3), fermented for 0, 12, and 24 h with *Lactiplantibacillus plantarum* ATCC 8014. Results are represented as mean values ± standard deviation (SD); *n* = 3.

The pH and TTA are two important indicators in monitoring the fermentation progress. The pH of the four types of sourdough with ancient and common flours started from a value slightly above a 6 at the moment of inoculation and reached, in the next 24 h, values below a 4. Respectively, the lowest pH was recorded in sample M_2_ (spelt flour sourdough) at 3.84, which was closely followed by sample M_1_ (einkorn flour sourdough) at 3.85; the pH differences between the samples were not found statistically significant (*p* < 0.05) (Figure 2). In the case of common wheat sourdough (M_0_), the pH value after 24 h of fermentation was 3.89.

Similar values were recorded by Casado et al. [78] for sourdough with wheat flour (3.9) fermented for 24 h at 35 °C. Regarding ancient wheat flours, a study carried out on einkorn flour sourdough fermented with different strains of LAB showed a pH decrease from 6.18 to 3.81 after 4 days of fermentation [68]. Additionally, the pH of the sourdough with spelt flour recorded in the first 24 h values between 4 and 5, as it was reported by [79]. Emmer wheat bran was part of a study that revealed that after 24 h of fermentation by *Lb. plantarum* T6 B10 and *Weissella confusa* BAN8, the pH of the obtained dough reached the 3.9 value [80].

According to Arora et al. [2], depending on the type of flour and the protocol used, the pH of sourdough is most often between 3.4 and 4.9. Values below 3 were normally recorded only in cases where the fermentation took longer than 48 h or if other special ingredients were used in the composition of the sourdough, such as brewer’s spent grains.

The pH value is influenced by the amount of acids formed during fermentation [68].

The total titratable acidity (TTA) helps to measure the total acids produced by *Lb. plantarum* ATTC 8014. On the other hand, TTA is considered an important indicator regarding acid flavor characteristics of sourdough because the production of lactic acid (the main metabolite of fermentation) has great relevance in terms of the aroma and shelf life of the final product [81].

In this case, it increased proportionally with the increase of fermentation time, reaching values of 15.6 mL of 0.1 N NaOH/10 g for the emmer sample (M_3_), and 19.8 mL of NaOH/10 g for the spelt sample (M_2_) after 24 h of fermentation at 35 °C. Einkorn (M_1_) and wheat flour (M_0_) led to an acidification rate of 23.2 and 23.4 mL of NaOH/10 g, the differences between these two samples not being statistic significative (*p* < 0.05) at the end of fermentation.

Values of total titratable acidity equal to 22.3 mL NaOH 1 N/100 g for wheat flour sourdough (after 24 h) [82] and around 25 mL 0.1 N NaOH/10 g for sourdough with rye and spelt flour (after 3 days) had been reported [83].

According to Arora et al. [2], the most common interval for TTA is established between 4.0 and 25.0 mL of 0.1 M NaOH/10 g of dough. The highest values being specific for sourdough fermented by heterofermentative bacteria [84].

The acidity value can be influenced by the metabolic activities of the bacteria, and affected by proteolysis, lipolysis, and amylolysis that occur during fermentation [85]. It is not without interest to mention that for this study, whole meal flours were used, which contribute to these acidification rates. The less refined a flour is, the higher its ash content (flour mineral content) [86], and according to Clément et al. [74], ash content shows a positive effect in terms of carbon dioxide production and acidity in the sourdough. Higher fermentation activity that occurs in the bread with high ash content sourdough leads to obtaining products with an increased volume and implicitly a lower density [74]. The whole meal flours used in the study had the following ash contents, according to the producers: 1.88% wheat, 2.48% einkorn, 1.65% spelt flour, and 1.50% emmer flour, which correlated with the determined acidity and supported this conclusion.

### 3.3. Carbohydrates and Organic Acids Content

*Lb. plantarum* ATCC 8014 induced a heterofermentative metabolism in common and ancient wheat sourdoughs, as can be seen in Table 2. The glucose content had an upward evolution after 24 h in the case of the 3 sourdough samples with ancient wheat flour (M_1_/einkorn—4.99 mg/g, M_2_/spelt—5.36 mg/g, M_3_/emmer—2.71 mg/g), and a downward evolution in the case of the control sourdough (common wheat flour—1.47 mg/g). The fructose content decreased after 24 h of fermentation in all samples, and a valid explanation could be given by its conversion to mannitol by mannitol dehydrogenase, as previously specified, but also due to its use as an alternative external electron acceptor by the lactic acid bacteria [87]. Due to the conversion of maltose to glucose and consumption during the fermentation process [29], the concentration of maltose decreased significantly in the first 3 samples after 24 h of fermentation (M_0_—1.39 mg/g, M_1_—1.11 mg/g, M_2_—1.875 mg/g). According to De Vuyst et al. [87], the use of maltose as the main source of energy through a dedicated catabolic pathway is characteristic of lactic acid bacteria that can be used in sourdough fermentation.

In Table 2 it can be observed that the sourdough with the einkorn flour (M_1_) presented higher values of glucose (4.99 mg/g) and fructose (2.07 mg/g) after 24 h of fermentation compared to the sourdough with common wheat (M_0_). A similar situation was described by other researchers, who reported that sourdough bread with einkorn organic flour presents a higher amount of carbohydrates (53.03 mg/100 g) than sourdough bread with wheat commercial flour (51.70 g/100 g), and sourdough bread with wheat organic flour (51.76 g/100 g) [88].

Our results are supported by a study conducted by Zörb et al. [89], who showed that spelt whole meal wheat flour compared to wheat whole meal flour is richer in free sugars, such as maltose (2.35 mg/g vs. 1.37 mg/g), fructose (0.36 mg/g vs. 0.17 mg/g), glucose (0.36 mg/g vs. 0.15 mg/g), sucrose (7.47 mg/g vs. 5.91 mg/g), or 1-kestose (3.08 mg/g vs. 2.00 mg/g).

Pozzo et al. [90] reported in the case of spelt flour fermented with a sourdough starter (Lievitamente SNC, Viareggio, Lucca, Italy) at the time of 0 for fermentation, the following values: glucose 1.74 mg/g, maltose 7.64 mg/g, fructose 3.17 mg/g, and sucrose 4.04 mg/g; and after 24 h of fermentation—glucose 15.37 mg/g, maltose 12.66 mg/g, 22.16 fructose mg/g, and sucrose 0.65 mg/g. Additionally, after another 24 h, except for maltose (7.77 mg/g), all other carbohydrates recorded values below 1 mg/g sample.

The differences between the maltose, glucose, and fructose consumption during fermentation of einkorn, emmer, and spelt comparing to common wheat are due to their higher content in starch and free simple sugars.

It was also reported that emmer sourdough revealed higher amounts of glucose and fructose than spelt or common wheat sourdough [57].

Regarding organic acids, these are products of lactic fermentation, with lactic acid being the most prevalent and significant of them, even if citric acid was present in unfermented flours in relative high amount. A variety of factors, including metabolic activity, technological performance, and sourdough’s acidification properties, influence the quantity of the acids produced [91]. Organic acids also play an important role in terms of the rheological properties of the dough. Particularly, lactic acid is recognized for the elastic structure it gives to the dough, while the acetic acid, on the contrary, leads to the formation of a harder gluten. Other benefits that are attributed to organic acids are their ability to protect products, from the point of view of microbiological safety [91].

In Table 3, a progressive increase in lactic acid concentration can be observed along with the increase in fermentation time. The highest values of lactic acid were recorded for samples M_0_—control sample, (6.65 mg/g), and M_1_—einkorn flour (6.36 mg/g), with the opposite pole being the sample with M_3_—emmer flour (2.89 mg/g).

Acetic acid is a minor product of heterofermentative metabolism and recorded maximum values of 1.09 mg/g in common wheat sourdough (M_0_), the most satisfactory concentrations being determined in samples with wheat and einkorn; its accumulation in sourdough being conditioned by the starter, flour type, and fermentation conditions [91].

Two major types of metabolic pathways act in the biosynthesis of aromatic substances in bread: the citric acid cycle and the amino acid metabolism. The first of them assumes that lactic acid bacteria are able to produce acetoin, diacetyl, butanediol, and other compounds in the process of metabolizing citrate [92]. In Table 2 samples M_1_ with 2.95 mg/g and M_2_ with 2.66 g/mg are significantly different (*p* < 0.05) from the wheat sample (M_0_) in terms of citric acid content, which can lead us to form the hypothesis that einkorn and spelt flours represent plant matrices that support the synthesis of citric acid by *Lb. plantarum* ATCC 8014. From a metabolomic point of view, this could be an important finding since specific aromatic compounds are formed as a result of the interaction between the raw flour and the LAB strain, and so the sensorial characteristics could be influenced.

In contrast with homofermentative lactic acid bacteria that only produce lactic acid, heterofermentative lactic acid bacteria also produce, among other compounds, ethanol [93]. In this study, ethanol showed low levels in all varieties of sourdough, and in some it was even imperceptible. However, compared to the start of fermentation, a slight accumulation can be observed in the samples after 24 h, as a result of glycolysis and the decomposition of pyruvate. The highest value was recorded in the sample with wheat flour (M_0_)—0.28 mg/g and emmer flour (M_3_)—0.12 mg/g. Two of the most important advantages that the accumulation of ethanol in sourdough brings are that it helps to strengthen the gluten network and that, according to Pérez-Alvarado et al. [94], ethanol and lactic acid isomers (at pH 4) can cause an increase in the metabolic activity of LAB.

Shewry et al. [57] reported higher values for lactic and organic acids in emmer and spelt sourdoughs fermented with a commercial starter culture compared to bread wheat, but the differences were not found to be significant. Novotni et al. [95] determined that in whole meal wheat sourdough fermented with *Lb. plantarum* DSM 2601 until a pH value of 4 was reached, lower concentrations of lactic acid (0.96 g/100 g) and acetic acid (0.01 g/100 g). This accumulation of acids was probably influenced by a lower time of fermentation. However, more close concentrations were reported by Ventimiglia et al. [96] when fifteen durum wheat sourdough samples were fermented with 28 strains of *Lb. plantarum*, ranging between 1.97and 9.41 mg/g lactic acid and 0.36 and 1.46 mg/g acetic acid at pH varying from 3.81 to 4.60.

Because the flavor of bakery products is greatly influenced by the organic acids that are formed during fermentation; the quotient of fermentation (QF) which represents the molar ratio between lactic and acetic acid, is a common and widely used parameter to correlate acidity and aroma. Most often, it is recommended to keep it at a value below 5 [2], or below 4 according to Coda et al. [28], when emmer and spelt sourdoughs were discussed. In this study, however, most of the samples registered a quotient of fermentation beyond these limits; the highest values were obtained after 24 h in the samples with einkorn flour (14.81) and the one with spelt flour (13.24). An explanation for these values is provided by Casado et al. [78] who note that a high fermentation temperature, such as 35 °C, facilitates microbial activity and implicitly increases the quotient of fermentation. Higher values of this parameter were also reported in other studies made on wheat sourdough: 15.64 in Galli et al. [97] research and 9.3 in Lattanzi et al. [98]. The molar ratio between lactic and acetic acids (fermentation quotient—QF) is greatly influenced by the ratio of dough yield and fermentation temperature. Thus, for the accumulation of acetic acid in larger quantities, temperatures between 25 and 30 °C are suitable, while lactic acid prefers temperatures of 35–37 °C [3], as they were set in the present case.

### 3.4. Crude Cellulose Content

Fiber solubilization is one of the most important processes during sourdough fermentation. Thus, fibers change their physical and chemical properties depending on the degree of fermentation. The ratio between soluble and insoluble fibers can be modified as a result of enzymatic reactions; in sourdough, there are two types of enzymatic hydrolysis that fibers can suffer. The first case supposes that when the flour is hydrated, certain hydrolytic enzymes intrinsic to the grains are activated, an example being hemicellulases. In the second case, LAB releases enzymes with glycolytic activity that can also act on the fibers in the dough [99].

**Figure 3:**
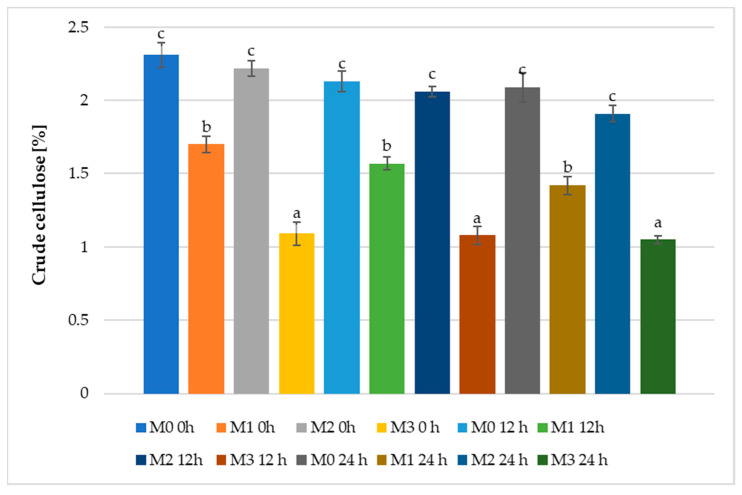
Crude cellulose in sourdough with wheat flour (M_0_), einkorn flour (M_1_), spelt flour (M_2_), and emmer flour (M_3_), fermented for 0, 12, and 24 h with *Lactiplantibacillus plantarum* ATCC 8014. Results are represented as mean values ± standard deviation (SD); *n* = 3. Different small letters show the significant difference (*p* < 0.05) between samples at the same moment (0, 12, 24 H).

Based on this information, it can also be observed (Figure 3) in the present case, there is a gradual decrease in the concentration of cellulose with the increase in the fermentation time of the sourdough. Thus, the sample with common flour (M_0_) had at time 0 h of the fermentation a cellulose value of 2.31%, which after 24 h decreased to 2.09%; sourdough with spelt flour (M_2_) left at 2.06% and arrived at 1.91% cellulose in the final, while the smallest amount was found in the emmer flour sample (M_3_), from 1.09% to 1.05%.

Until now, the amount of research occurring on ancient cereals has been quite limited, making it difficult to make comparisons and adopt unanimously accepted opinions regarding their chemical composition, and especially their crude cellulose content. However, according to Kulathunga et.al. [100] emmer flour has an insoluble fiber content between 7.8 and 13.8%, spelt flour has 10.6–11.4%, and einkorn flour has 6.9–7.53%. The same authors recorded in the breads produced from these flours the following concentrations in terms of total insoluble fibers: 8.1–8.4% einkorn flour bread, 7.6–8.1% spelt flour bread, and 7.2–7.3% emmer flour bread [100]. A slight increase in the percentage of soluble fibers was reported, which, was attributed to the solubilization of insoluble fibers occurring during the fermentation or baking processes [100]. On the other hand, KAMUT^®^ Khorasan (another ancient wheat species) flour bread with sourdough fermented at low temperature recorded 13.26 g/100 g insoluble fibers, while the same type of bread obtained with sourdough fermented at high temperature had a value of 18.11 g/100 g. These results demonstrate once again that the enzymatic processes during fermentation and baking are an important factor in terms of the functional properties of the final product [101].

The addition of cellulose to bakery products to increase their total fiber content has been the subject of several studies [102,103,104,105,106]. In principle, the consumption of dietary fiber is associated with the prevention or treatment of various diseases [107,108,109]. The insoluble fibers, including cellulose, which are mainly found in cereals, have revealed certain health benefits such as reduced blood sugar, prevention of cardiovascular risks and coronary artery disease, growth of intestinal peristalsis, decreased contact time between toxic compounds and intestinal mucosa, speeding up intestinal transit, helping in the detoxification process, and weight loss [110,111,112].

### 3.5. Minerals Content

The importance of minerals in human health is well known and has been widely demonstrated through a series of studies [113,114,115]. The sources from which they can be procured are various, with the largest quantities being found in milk, dairy products, green leafy vegetables (spinach, cabbage, kale), broccoli, citrus fruits, kiwis, and bananas [116]. In addition, cereals and cereal products also contain important quantities of iron, zinc, manganese, phosphorus, and sodium [117].

Additionally, the increase in the bioavailability of minerals through sourdough fermentation has been supported by several researchers [99,118,119]. The mechanism underlying this process is related to the acidification of the sourdough; which in an indirect way activates the endogenous phytases of the cereal, as well as microbial enzyme activities [2]. Phytic acid/phytate is a substance that is naturally found in the aleurone layer of grains and that exhibits a strong chelating capacity, it also affects the absorption of minerals in the body by forming insoluble complexes with dietary cations [120]. In the case of increasing bioavailability of macro and micronutrients, the optimal pH for acidification must be between 4.3 and 4.6 and phytic acid must drop above 70% [2].

Regarding the present study, the mineral content (Table 4) of the four varieties of sourdough increased with the increase of the duration of fermentation. Einkorn flour sourdough (M_1_) stood out for its high content in calcium—246 mg/kg (Ca), zinc—36 mg/kg (Zn), manganese—46 mg/kg (Mn), and iron—19 mg/kg (Fe) reached after 24 h of fermentation [121]. The sample with spelt flour (M_2_) was highlighted by the significant magnesium (Mg) content of 155 mg/kg, which is similar to that of the wheat flour sourdough (M_0_). The lowest values for most minerals were determined in the sample with emmer flour (M_3_), but this can be explained by the fact that this cereal has a lower content of minerals such as zinc (22.8 mg/kg), iron (34.1 mg/kg), calcium (360 mg/kg), and manganese (24 mg/kg) [31,122] compared to einkorn flour—M_1_ (Zn—54.8 mg/kg, Fe—47 mg/kg, Ca—420 mg/kg, Mn 49.3 mg/kg) [40], spelt (Zn—22.9 mg/kg, Fe—45.9 mg/g, Ca—390 mg/kg, Mn—27 mg/kg) [31,122], and wheat (Zn—34.6 mg/kg, Fe—37.5 mg/g, Ca—430 mg/kg, Mn—26 mg/kg) [122]. According to Zahra et al. [123], the wheat dough fermented for 6 h with *Lb. plantarum* E90 registered an increase from 3.08 (0% culture dose) to 8.95 mg/kg (2% culture dose) in terms of iron content, and from 3.45 (0% culture dose) to 11.04 mg/kg (2% culture dose) in the case of zinc content.

The mineral content of the sourdough is closely related to the initial content of the flour used, which is in turn influenced by several factors related to the growth and development of the plant. According to Spisni et al. [124], both in the case of ancient and modern varieties of wheat, the mineral content is influenced by several variables such as climate, soil type, and geographical area.

The consumption of minerals is essential for a healthy body because they perform different metabolic functions; for example, calcium has a role in blood coagulation, sodium helps to decrease blood pressure, magnesium is involved in muscle relaxation, and zinc acts in protein synthesis [125]. Therefore, the production of functional foods with a high nutritional value is a necessity, and the utilization of materials rich in minerals and bioactive compounds, such as ancient flours, represent the first steps in this direction [46].

### 3.6. Volatile Compounds Content

The sensorial quality of bakery products is significantly influenced by their aromatic profile. According to Pétel et al. [126] over the years, more than 500 volatile compounds were identified in bread. On the other hand, in sourdough and sourdough bread, only around 200 compounds have been identified, with the studies on these being in a much smaller number [127]. In sourdough products, the lactic bacteria (LAB) are the ones that form the basis of the generation of volatiles, while factors that condition their activity like water content and temperature are responsible for the amount formed [128]. Normally, lactobacilli carry out the acidification of the product and also, releases flavor precursors such as free amino acids that increase during sourdough fermentation [128].

Cereals contain a wide variety of specific volatile compounds, which, depending on the type and concentration in which they are found, form the olfactory perception [129]. Their formation is conditioned by certain factors such as pH, amino acid profile, sugar profile, heating temperature, and time [129]. Chai et al. [130] identified over 90 volatiles in wheat flour. Most of them are from the class of aldehydes, and contain volatiles such as hexanal, nonanal, 3-methyl-butanal, heptanal, octanal, and (E)-2-nonenal which are also the most involved in the development of the bread profile aroma. Among the ketones, the following stood out: 2,3-butanedione (the most relevant compound present in bread), 2,3-Pentanedione, and 6-methyl-5-hepten-2-one; and the class of furans includes acetophenone, benzaldehyde, and furfural.

Sourdough—made with common wheat or with ancient wheat—also, contains a number of typical volatile compounds such as pental, hexanal, 5-methyl-3-hexanone, 1-pentanol 2-octenal, acetoin, furan 2,6-dimethyl-4-heptanone, octyl acetat, diacetyl, 4-methyl-3-penten-2-one, 6-methyl-5-hepten-2-one, as reported in other research studies [26,126,131].

In the present study, a total of 43 aromatic compounds were identified and classified into alcohols, aldehydes, ketones, acids, and other compounds (Table 5). From the class of alcohols, the most representative was 1-Hexanol with values (after 24 h) between 11.74% (M_0_—wheat flour sourdough) and 69.5% (M_3_—emmer flour sourdough) of the total surface of the peaks; this was perceived as having a delicate fatty-fruity, fermented, and woody profile. It has also been reported in other studies as one of the most abundant alcohols [126,132], and together with its aldehyde (hexanal), they are most abundant in the bread loaf (without sourdough) [7]. From the group of aldehydes (except hexanal), benzaldehyde was the compound identified in all samples, at the end of the fermentation process registering the highest percentage in the sample with einkorn flour (2.80%) and wheat flour (1.17%). This compound had a spicy, almond flavor, and it is formed in the dough in two ways, the degradation of amino acids or autoxidation of 2,4-decadienal [131]. Regarding ketones, mainly acetophenone (0.18–7.53%) and 2-heptanone (0.3–10.4%) were identified. These degrade with increasing fermentation time, but are considered to be important indicators regarding the freshness of the sourdough and the final product [131]. Acids were found in a low proportion, among them caproic acid/hexanoic acid, which were recognized for their ability to inhibit the growth of fungi [133]. Of the rest of the reported compounds, 2-pentylfuran, limonene, d-limonene, and butanoic acid, ethyl ester deserves to be highlighted. They were also identified in other studies and are recognized for the sweet, fruity aroma they give to the dough [126,134,135]. The presence of compounds with a less pleasant aroma, such as dimethyl disulfide and dimethyl trisulfide was also observed, but in small amounts and mainly in the wheat flour samples—M_0_.

In support of these results comes a study conducted by Starr et al. [138] who identified 88 compounds in wheat varieties, such as hexanal, hexanol, 2-pentylfuran, benzaldehyde, 2-methylbutanal, 3-methyl-1-butanol, 6-methyl-5-heptene- 2- one, 2-methyl-1-butanol or 2-nonenal. As can be seen in Table 4, there is a decrease in some volatile compounds with the increase of the fermentation time; this trend was also confirmed in another study, where during the fermentation of sourdough made with whole wheat there was registered a decrease in the concentration of ketones, aldehydes, and heterocycles [131].

However, the results obtained show a wide range of volatiles that form a complex aromatic profile. Their importance to the final product is major in the sense that they help to establish the degree of acceptability of the product by consumers.

### 3.7. Rheological Values

**Figure 4:**
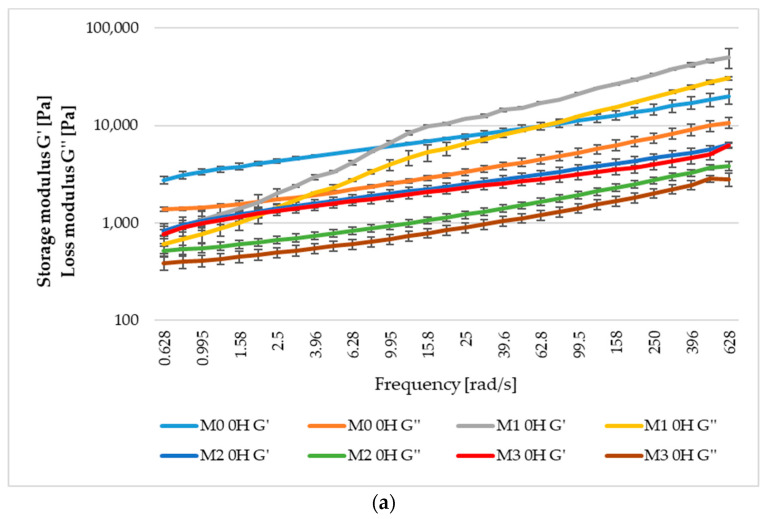

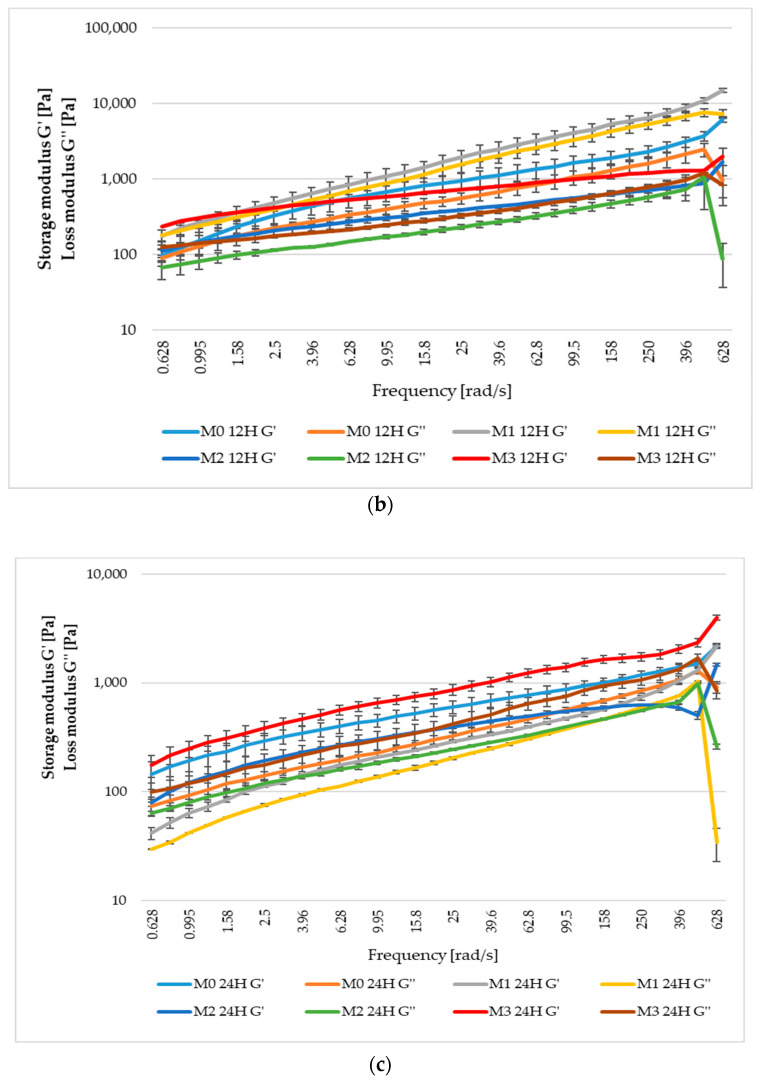
The storage (G’) and loss (G’’) modulus for sourdough fermented for 0 (**a**), 12 (**b**), and 24 (**c**) hours with *Lactiplantibacillus plantarum* ATCC 8014.

The rheological properties of the sourdough with wheat, einkorn, spelt, and emmer are presented in Figure 4; the storage modulus (G’) and the loss modulus (G’’) were measured for the three fermentation times (0, 12, 24 h) at an angular frequency between 0.628 and 628 rad/s. These two moduli are the most often used to characterize the dynamic properties of the dough; the first, respectively, the storage modulus indicates the materials’ capacity to store elastic deformation energy, while the second, the loss modulus, indicates the viscous portion of the materials [66].

Mainly, the two moduli grew with the increase of the angular frequency, a fact that can be explained by the increase in the structure of the sourdough [139]; also, G’ was higher than G’’ which means that the dough has an elastic behavior. After 24 h of fermentation at an angular frequency of 628 rad/s, the highest value for storage modulus (G’) was recorded in the case of sample M_3_ with 3988.9 Pa, and for loss modulus (G’’) the major value was 915.8 Pa in sample M_0_. Additionally, the two moduli registered decreases during the 24 h for all flour variants, a possible reason for this reduction being the pH, namely, sourdough acidification affects chemical compounds, thus improving the interactions between water molecules and structural components like starch and proteins [139].

Hadnađev et al. [140] studied the rheological properties of some of the types of flour obtained from ancient cereals and came to the conclusion that they are influenced to a large extent by the quantity and quality of wheat gluten. Thus, compared to spelt, emmer flour presented a high wet gluten value and a low gluten index thus producing a dough with an elastic structure that was able to generate and retain the largest amount of gas during fermentation, but also quickly collapsed and lost its structure, releasing the most quantity of carbon dioxide into the atmosphere.

Fermentation has a significant impact on the rheological properties of the dough, being in turn influenced by the species of microorganisms, their metabolic activity, and the pH value that develops over time [141]. There are a number of papers that have studied the influence that the water content has on the rheological properties of the dough [142,143,144]. In the present study, proteolysis, which occurs during the fermentation process and lowers the starch level, can be the cause of the decrease in sourdough viscosity and elasticity [141].

### 3.8. Effect of Sourdough Type on Metabolic Profile

**Figure 5:**
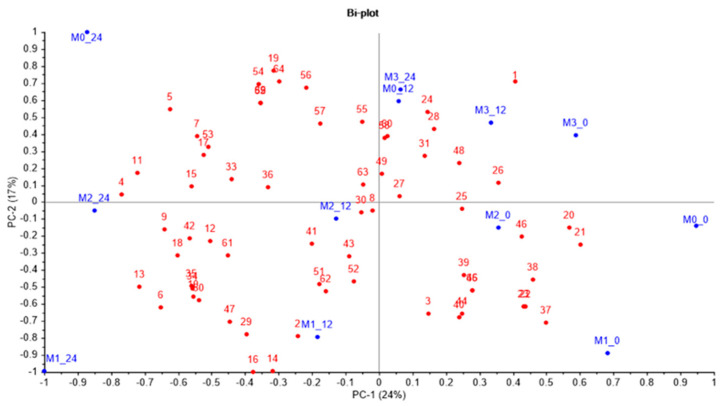
Principal Component Analysis (PCA) biplot of the compounds and parameters identified in sourdough fermented for 0, 12, and 24 h with *Lactiplantibacillus plantarum* ATCC 8014. (1—Maltose; 2—Glucose; 3—Fructose; 4—Lactic acid; 5—Acetic acid; 6—Citric acid; 7—Ethanol; 8—Cellulose; 9—K; 10—Ca; 11—Mg; 12—Cd; 13—Cu; 14—Zn; 15—Cr; 16—Mn; 17- Ni; 18—Fe; 19—Pb; 20—G’ 0.628; 21—G’’ 0.628; 22—G’628; 23—G’’ 628; 24—1—Butanol, 3—methyl—; 25—1—Pentanol; 26—1—Pentanol, 4—methyl—; 27—1—Hexanol; 28—1—Octen—3—ol; 29—1—Butanol, 2—methyl—; 30—1—Nonen—3—ol; 31—1—Heptanol; 32—1—Penten—3—ol, 4—methyl—; 33—2—Octen—1—ol; 34—3—Buten—1—ol, 3—methyl—; 35—1—Butanol, 3—methyl—; 36—2—Hexanol, 5—methyl—; 37—Hexanal; 38—Heptanal; 39—Benzaldehyde; 40—Octanal; 41—2—Heptenal, (Z)—; 42—Benzeneacetaldehyde; 43—2—Octenal, (E)—; 44—Nonanal; 45—Decanal; 46—Acetophenone; 47—2-Heptanone; 48—3—Octen—2—one; 49—2—Butanone, 3—hydroxy—; 50—2—Nonanone; 51—Butanoic acid; 52—Hexanoic acid; 53—Benzoic acid; 54—Butanoic acid, ethyl ester; 55—Butanoic acid, propyl ester; 56—Butanoic acid, butyl ester; 57—Hexanoic acid, ethyl ester; 58—Acetic acid, hexyl ester; 59—1-Butanol, 3-methyl—, acetate; 60—Acetic acid, pentyl ester; 61—Furan, 2—pentyl—; 62—Limonene; 63—D—Limonene; 64—Disulfide, dimethyl; 65—Dimethyl trisulfide; 66—n.i).

**Figure 6:**
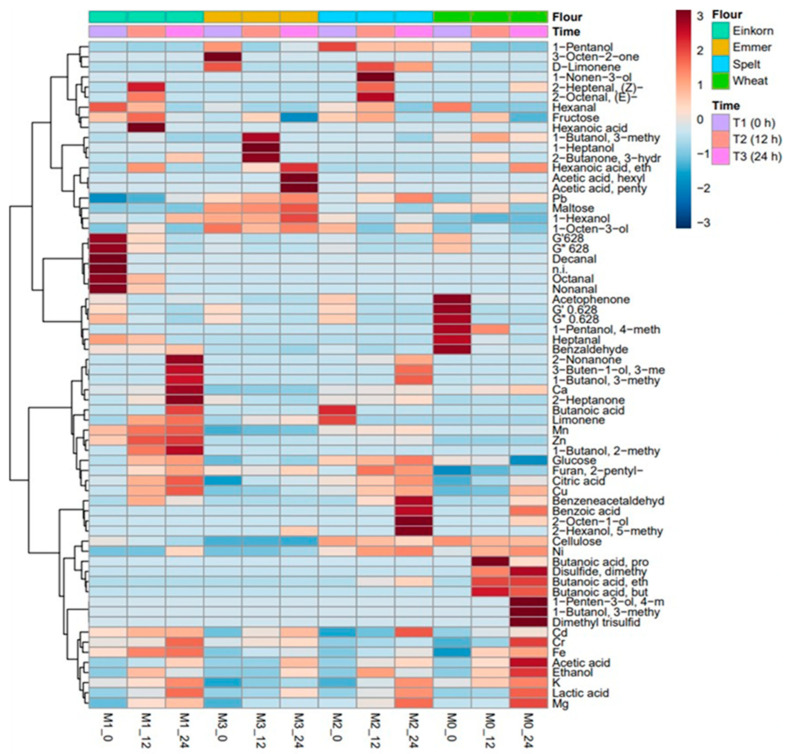
Heat map and HCA of the compounds and parameters identified in sourdough fermented for 0, 12, and 24 h with *Lactiplantibacillus plantarum* ATCC 8014. Where, the colors represent the compounds’ level, starting from light blue (low level) to white to dark red (high level).

Based on the 66 parameters determined in this experiment (see Figure 5 legend), after a weighted standard deviation pre-treatment applied for providing a relative significance to each value, a Principal Component Analysis (PCA) was performed. Principal component Analysis (PCA) and cluster analysis using a heatmap (Figure 6) were used for a deeper investigation of the effect of sourdough type (ancient wheat flour type) and fermentation moment on their metabolic profiles. As the PCA plot shows (Figure 5), the two principal components (PC-1, PC-2) and their scores explain 24% and 17% of the range in data variation. The plot indicates a clear separation of the ancient wheat flour (0 h of fermentation) from the sourdoughs after 12 and 24 h of fermentation. It also shows a clear distinction of the sourdoughs obtained with wheat (M_0_), einkorn (M_1_), and spelt (M_2_) after 24 h of fermentation from the other samples. In the case of emmer (M_3_) flour, the separation between the samples at 0, 12 and 24 h of fermentation exists, but is smaller, indicating that emmer flour had a different behavior during sourdough fermentation. This aspect could be observed from the heatmap too (Figure 6), indicating that maltose, glucose, and fructose were metabolized differently in emmer flour than in the other flours. This behaviour could be explained by a higher content of starch in emmer compared to einkorn and spelt, but also due to its high content of resistant starch [145,146]. Moreover, the lactic acid concentration was smaller during the emmer sourdough fermentation in comparison to the rest of the flours. It is also possible that the smaller amounts of certain minerals in emmer, which are important for the microbial cell development, negatively influenced the *Lb. plantarum* ATCC 8014 metabolism. The volatile profile of emmer sourdough showed differences as a result of the microbial activity. The volatiles formed in the highest concentration (dark red) were: 1 butanol-3 methyl, 1-heptanol, 1 hexanol, 2 butanone-3 hydroxy, hexanoic acid, acetic acid, hexyl ester and acetic acid, pentyl ester. Inside the cluster indicates a grouping of these volatile compounds, also.

As indicated on the heatmap, three aldehydes, decanal, octanal, and nonanal were in the highest concentrations in the einkorn sourdough at the inoculation moment. Their level decreased during fermentation. The specific microbial volatile metabolites that formed in the highest amounts as a result of the interaction between einkorn flour and *Lb. plantarum* metabolism were: 2-nonanone, 3-buten-1-ol, 3-methyl, 1-butanol, 3-methyl, 2-heptanone, 1-Butanol, 2-methyl. HCA analysis indicated a clear the clusterisation of these volatile derivatives. Common wheat flour was characterized by a group of volatiles (dark red) composed of: acetophenone, 1-pentanol-4-methyl, heptanol, and benzaldehyde. The volatiles profile during fermentation showed increasing levels of butanoic acid, butanoic acid, ethyl ester, butanoic acid, propyl ester, disulfide, dimethyl, dimethyl trisulfide, 1-butanol, 3-methyl and 1-Penten-3-ol, 4-methyl, and within the cluster their grouping is evident. Spelt flour sourdough reveals increased levels of 1-butanol-2 methyl, furan-2 pentyl, citric acid, benzene-acetaldehyde, benzoic acid, 2 octen-1-ol, 2-hexanol-5 methyl. These volatile compounds could be found grouped within the cluster. The levels of K, Mg, Fe also showed increasing levels during the fermentation period in all flours except emmer sourdough. Mn and Zn were grouped within the cluster and showed the highest amounts in einkorn sourdough; this could explain the great support of einkorn flour for the *Lb. plantarum* cell development since these minerals are co-factors for enzymes.

The rheological parameters G’ and G’’ also showed a clear separation between the sourdough samples. The storage modulus (G’) and the loss modulus (G’’) revealed a distinct rheological profile for common wheat sourdough than in the case of the ancient wheat sourdoughs. This behavior is due to its higher glutenin (GLUT) contents compared to einkorn, emmer, and spelt, resulting in increasing ratios of gliadin to glutenin (GLIA/GLUT), as reported by [147]. High glutenin content and a low ratio of GLIA/GLUT are the two parameters influencing the dough quality. However, the decreasing values of the two moduli during the fermentation of einkorn, spelt, and emmer (used in this study) indicate good rheological behavior suitable for good baking performance.

## 4. Conclusions

According to the obtained results, it can be stated that *Lactiplantibacillus plantarum* ATCC 8014 shows good adaptability, with a high cell count and a good acidification rate, in the sourdough obtained with wheat flour, einkorn flour, spelt flour, and emmer flour. The metabolic profiles of the ancient wheat and common wheat sourdoughs indicated clear differences between them. It was possible to mark out specific metabolites as a result of the interaction between ancient wheat flours and *Lb. plantarum* ATCC 8014. From the ancient wheat, emmer showed distinctive behaviour during fermentation in terms of cell dynamics, sugar metabolization, lactic acid formation. This could be explained by its higher content in resistant starch, but also due to the smaller amounts in Zn and Mn, important factors for the microbial cell propagation.

The cluster analysis showed specific volatile compounds for each type of sourdough. Moreover, by this approach, it is possible to identify volatile derivatives with pleasant or unpleasing odours resulting from the interaction between the raw flour and the bacterial strain. It could be facilitating the setation of some desired sensorial characteristics in terms of flavour to obtain whole-meal breads with a higher degree of consumer acceptability.

Future studies will be conducted on using emmer, einkorn, and spelt sourdough as biocarrier of nutritional valuable compounds (fibers, minerals, amino acids, etc.) to obtain bread assortments with low glycemic index and improved health benefits.

## Figures and Tables

**Table 1:** Sourdough formulations for wheat, einkorn, spelt, and emmer flour and sample codifications.

Materials	M_0_	M_1_	M_2_	M_3_
Wheat wholemeal four, g	500	-	-	-
Einkorn wholemeal flour, g	-	500	-	-
Spelt wholemeal flour, g	-	-	500	-
Emmer wholemeal four, g	-	-	-	500
*Lactiplantibacillus plantarum* ATCC 8014 suspension, mL	100	100	100	100
Water, mL	400	400	400	400

where: M_0_—wheat flour sourdough; control sample, M_1_—einkorn flour sourdough, M_2_—spelt flour sourdough, M_3_—emmer flour sourdough.

**Table 2:** Carbohydrates content (maltose, glucose, fructose) in sourdoughs with wheat flour (M_0_), einkorn flour (M_1_), spelt flour (M_2_), and emmer flour (M_3_), fermented for 0, 12, and 24 h with *Lactiplantibacillus plantarum* ATCC 8014.

Sample	Hours of Fermentation [H]	Maltose [mg/g]	Glucose [mg/g]	Fructose [mg/g]
M_0_	0	5.807 ± 0.09 ^c^	3.689 ± 0.06 ^b c^	1.76 ± 0.09 ^a^
M_1_		1.393 ± 0.02 ^a^	3.025 ± 0.03 ^b^	2.865 ± 0.07 ^b^
M_2_		3.552 ± 0.04 ^b^	4.251 ± 0.08 ^c^	2.774 ± 0.04 ^b^
M_3_		8.776 ± 0.08 ^d^	2.252 ± 0.05 ^a^	1.804 ± 0.09 ^a^
M_0_	12	6.465 ± 0.03 ^c^	3.489 ± 0.08 ^a^	2.861 ± 0.05 ^a b^
M_1_		1.695 ± 0.04 ^a^	4.527 ± 0.07 ^a^	3.821 ± 0.06 ^c^
M_2_		3.347 ± 0.07 ^b^	4.63 ± 0.05 ^b^	3.183 ± 0.08 ^b^
M_3_		9.362 ± 0.06 ^d^	3.096 ± 0.03 ^a^	2.669 ± 0.06 ^a^
M_0_	24	1.389 ± 0.02 ^a^	1.466 ± 0.05 ^a^	1.056 ± 0.08 ^a^
M_1_		1.113 ± 0.05 ^a^	4.986 ± 0.08 ^c^	2.065 ± 0.04 ^b^
M_2_		1.875 ± 0.01 ^a^	5.355 ± 0.09 ^c^	1.743 ± 0.06 ^b^
M_3_		11.339 ± 0.05 ^b^	2.709 ± 0.03 ^b^	0.513 ± 0.07 ^a^

where: Results are represented as mean values ± standard deviation (SD), mg/g, *n* = 3. Different small letters show the significant difference (*p* < 0.05) between M_0_ (wheat flour sourdough), M_1_ (einkorn flour sourdough), M_2_ (spelt flour sourdough), and M_3_ (emmer flour sourdough) at the same moment (0, 12, 24 H).

**Table 3:** Organic acids and ethanol content in sourdoughs with wheat flour (M_0_), einkorn flour (M_1_), spelt flour (M_2_), and emmer flour (M_3_), fermented for 0, 12, and 24 h with *Lactiplantibacillus plantarum* ATCC 8014.

Sample	Hours of Fermentation [H]	Lactic Acid [mg/g]	Acetic Acid [mg/g]	Citric Acid [mg/g]	Ethanol [mg/g]	QF
M_0_	0	0.00 ^a^	0.00 ^a^	1.35 ± 0.03 ^a^	0.00 ^a^	n.c.
M_1_		0.00 ^a^	0.00 ^a^	1.91 ± 0.03 ^b^	0.00 ^a^	n.c.
M_2_		0.00 ^a^	0.00 ^a^	2.09 ± 0.04 ^b^	0.00 ^a^	n.c.
M_3_		0.00 ^a^	0.00 ^a^	1.21 ± 0.02 ^a^	0.00 ^a^	n.c.
M_0_	12	0.40 ± 0.02 ^a^	0.36 ± 0.04 ^b^	1.72 ± 0.03 ^a^	0.14 ± 0.05 ^a^	1.11
M_1_		1.85 ± 0.03 ^b^	0.14 ± 0.02 ^a^	2.50 ± 0.03 ^b^	0.16 ± 0.02 ^a^	13.21
M_2_		1.89 ± 0.04 ^b^	0.27 ± 0.02 ^b^	2.33 ± 0.05 ^b^	0.19 ± 0.01 ^a^	7.00
M_3_		0.41 ± 0.02 ^a^	0.00 ^c^	1.82 ± 0.03 ^a^	0.00 ^b^	0.00
M_0_	24	6.65 ± 0.06 ^c^	1.10 ± 0.03 ^b^	2.00 ± 0.04 ^a^	0.28 ± 0.03 ^a^	6.05
M_1_		6.37 ± 0.02 ^c^	0.43 ± 0.05 ^a^	2.95 ± 0.03 ^b^	0.08 ± 0.02 ^a^	14.81
M_2_		5.43 ± 0.07 ^b^	0.41 ± 0.03 ^a^	2.66 ± 0.08 ^b^	0.08 ± 0.01 ^a^	13.24
M_3_		2.89 ± 0.03 ^a^	0.51 ± 0.02 ^a^	1.91 ± 0.07 ^a^	0.12 ± 0.03 ^a^	5.67

where: n.c.—not calculable. Results are represented as mean values ± standard deviation (SD), mg/g, *n* = 3. QF—quotient of fermentation (molar ratio between lactic and acetic acid). Different small letters show the significant difference (*p* < 0.05) between M_0_ (wheat flour sourdough), M_1_ (einkorn flour sourdough), M_2_ (spelt flour sourdough), and M_3_ (emmer flour sourdough) at the same moment (0, 12, 24 H).

**Table 4:** Micro and macronutrients in sourdough with wheat flour (M_0_), einkorn flour (M_1_), spelt flour (M_2_), and emmer flour (M_3_), fermented for 0, 12, and 24 h with *Lactiplantibacillus plantarum* ATCC 8014.

Minerals	0 h of Fermentation [H]				12 h of Fermentation [H]				24 h of Fermentation [H]			
[mg/kg]	M_0_	M_1_	M_2_	M_3_	M_0_	M_1_	M_2_	M_3_	M_0_	M_1_	M_2_	M_3_
**K**	346.93 ± 0.05 ^c^	347.3 ± 0.27 ^c^	334.22 ± 0.56 ^b^	332.86 ± 0.16 ^a^	353.76 ± 0.30 ^d^	350.63 ± 0.55 ^c^	345.87 ± 0.26 ^b^	338.730.34± ^a^	362.4 ± 0.14 ^b^	361.57 ± 0.15 ^b^	360.85 ± 0.15 ^b^	341.95 ± 0.15 ^a^
**Ca**	143.88 ± 0.04 ^b^	146.66 ± 0.33 ^c^	148.95 ± 0.11 ^d^	122.49 ± 0.34 ^a^	156.41 ± 0.23 ^c^	150.94 ± 0.21 ^b^	151.85 ± 0.30 ^b^	124.73 ± 0.20 ^a^	171.05 ± 0.17 ^c^	246.73 ± 0.30 ^d^	155.66 ± 0.20 ^b^	124.93 ± 0.12 ^a^
**Mg**	115.78 ± 0.08 ^ab^	114.99 ± 0.70 ^a^	115.84 ± 0.19 ^ab^	114.84 ± 0.21 ^a^	115.86 ± 0.37 ^a^	116.46 ± 0.20 ^a b^	116.26 ± 0.21 ^a b^	115.74 ± 0.20 ^a^	118.35 ± 0.35 ^c^	116.96 ± 0.17 ^b^	117.97 ± 0.17 ^c^	115.84 ± 0.19 ^a^
**Cd**	0.11 ± 0.07 ^a^	0.14 ± 0.03 ^a^	0.08 ± 0.02 ^a^	0.10 ± 0.03 ^a^	0.13 ± 0.05 ^a^	0.16 ± 0.05 ^a^	0.10 ± 0.03 ^a^	0.13 ± 0.11 ^a^	0.14 ± 0.05 ^a^	0.16 ± 0.05 ^a^	0.19 ± 0.04 ^a^	0.16 ± 0.05 ^a^
**Cu**	2.36 ± 0.09 ^a^	2.81 ± 0.15 ^a^	2.95 ± 0.14 ^a^	2.46 ± 0.14 ^a^	2.44 ± 0.26 ^a^	3.57 ± 0.29 ^b^	3.39 ± 0.17 ^b^	2.57 ± 0.12 ^a^	3.53 ± 0.11 ^b^	4.06 ± 0.10 ^bc^	3.59 ± 0.13 ^b^	2.79 ± 0.09 ^a^
**Zn**	14.89 ± 0.14 ^a^	24.85 ± 0.16 ^d^	18.74 ± 0.25 ^c^	16.84 ± 0.28 ^b^	14.90 ± 0.43 ^a^	34.20 ± 0.19 ^d^	18.96 ± 0.18 ^c^	16.98 ± 0.15 ^b^	15.36 ± 0.33 ^a^	36.07 ± 0.26 ^d^	19.85 ± 0.22 ^c^	17.23 ± 0.06 ^b^
**Cr**	0.18 ± 0.06 ^a^	0.38 ± 0.08 ^a^	0.25 ± 0.05 ^a^	0.35 ± 0.07 ^a^	0.27 ± 0.13 ^a^	0.39 ± 0.08 ^a^	0.29 ± 0.06 ^a^	0.4 ± 0.09 ^a^	0.69 ± 0.13 ^a^	0.63 ± 0.21 ^a^	0.32 ± 0.02 ^a^	0.43 ± 0.08 ^a^
**Mn**	21.75 ± 0.10 ^b^	34.61 ± 0.63 ^d^	25.83 ± 0.22 ^c^	10.20 ± 0.22 ^a^	22.34 ± 0.32 ^b^	43.99 ± 0.22 ^d^	27.62 ± 0.29 ^c^	12.69 ± 0.22 ^a^	22.86 ± 0.21 ^b^	46.70 ± 0.15 ^d^	28.95 ± 0.15 ^c^	13.85 ± 0.17 ^a^
**Ni**	0.35 ± 0.02 ^a^	n.d.	0.47 ± 0.10 ^a^	n.d.	0.75 ± 0.09 ^a^	n.d.	0.85 ± 0.09 ^a^	n.d.	0.90± ^a b^	0.57 ± 0.10 ^a^	0.94 ± 0.12 ^a b^	0.14 ± 0.04 ^a^
**Fe**	8.44 ± 0.03 ^a^	14.86 ± 0.19 ^c^	10.86 ± 0.23 ^b^	10.85 ± 0.23 ^b^	15.67 ± 0.19 ^b^	18.74 ± 0.16 ^c^	12.59 ± 0.19 ^a^	12.84 ± 0.17 ^a^	17.47± ^b^	19.29 ± 0.16 ^c^	13.96 ± 0.16 ^a^	13.46 ± 0.21 ^a^
**Pb**	5.46 ± 0.13 ^b^	4.14 ± 0.15 ^a^	6.49 ± 0.33 ^c^	7.50 ± 0.13 ^d^	6.77 ± 0.17 ^b^	4.9 ± 0.11 ^a^	7.48 ± 0.27 ^c^	8.24 ± 0.08 ^d^	7.29± ^a b^	6.70 ± 0.18 ^a^	8.93 ± 0.14 ^c^	8.94 ± 0.10 ^c^

where: n.d.—not detected. Results are represented as mean values ± standard deviation (SD), mg/kg, *n* = 3. Different small letters show the significant difference (*p* < 0.05) between M_0_ (wheat flour sourdough), M_1_ (einkorn flour sourdough), M_2_ (spelt flour sourdough), and M_3_ (emmer flour sourdough) at the same moment (0, 12, 24 H).

**Table 5:** Volatile compounds in sourdough with wheat flour (M_0_), einkorn flour (M_1_), spelt flour (M_2_), and emmer flour (M_3_), fermented for 0, 12, and 24 h with *Lactiplantibacillus plantarum* ATCC 8014.

Volatile Compounds	0 h of fermentation [H]				12 h of Fermentation [H]				24 h of Fermentation [H]			Odor Perception	
	M_0_	M_1_	M_2_	M_3_	M_0_	M_1_	M_2_	M_3_	M_0_	M_1_	M_2_	M_3_	
**Alcohols**													
1-Butanol, 3-methyl-	4.74 ± 0.07 ^c^	1.78 ± 0.11 ^a^	2.93 ± 0.09 ^b^	n.d.	12.33 ± 0.07 ^b^	3.82 ± 0.09 ^a^	3.42 ± 0.06 ^a^	23.52 ± 0.16 ^c^	7.04 ± 0.10 ^b^	n.d.	n.d.	2.77 ± 0.08 ^a^	acid, spicy
1-Pentanol	17.85 ± 0.13 ^b^	8.69 ± 0.12 ^a^	30.21 ± 0.23 ^d^	23.79 ± 0.12 ^c^	5.74 ± 0.08 ^a^	8.00 ± 0.04 ^b^	19.74 ± 0.08 ^c^	7.90 ± 0.11 ^b^	5.83 ± 0.17 ^a^	8.26 ± 0.15 ^b^	20.00 ± 0.04 ^d^	12.09 ± 0.05 ^c^	pungent
1-Pentanol, 4-methyl-	2.01 ± 0.07	n.d.	n.d.	n.d.	1.21 ± 0.09	n.d.	n.d.	n.d.	n.d.	n.d.	n.d.	n.d.	nuts
1-Hexanol	15.70 ± 0.09 ^a^	29.14 ± 0.17 ^b^	35.16 ± 0.19 ^c^	51.96 ± 0.17 ^d^	9.75 ± 0.11 ^a^	24.88 ± 0.12 ^c^	20.45 ± 0.10 ^b^	51.50 ± 0.15 ^d^	11.74 ± 0.09 ^a^	47.60 ± 0.15 ^c^	29.94 ± 0.07 ^b^	69.5 ± 0.10 ^d^	fruits
1-Octen-3-ol	n.d.	n.d.	2.27 ± 0.05 ^a^	3.08 ± 0.12 ^b^	0.93 ± 0.11 ^a^	1.48 ± 0.15 ^a b^	n.d.	2.22 ± 0.11 ^c^	n.d.	n.d.	1.91 ± 0.04 ^a^	2.93 ± 0.07 ^b^	mushrooms
1-Butanol, 2-methyl-	n.d.	n.d.	n.d.	n.d.	n.d.	0.93 ± 0.13	n.d.	n.d.	n.d.	1.35 ± 0.08	n.d.	n.d.	alcohol, wine
1-Nonen-3-ol	n.d.	n.d.	n.d.	n.d.	n.d.	n.d.	1.37 ± 0.09	n.d.	n.d.	n.d.	n.d.	n.d.	oil
1-Heptanol	n.d.	n.d.	n.d.	n.d.	n.d.	n.d.	n.d.	0.33 ± 0.04	n.d.	n.d.	n.d.	n.d.	woody, fatty
1-Penten-3-ol, 4-methyl-	n.d.	n.d.	n.d.	n.d.	n.d.	n.d.	n.d.	n.d.	1.16 ± 0.06	n.d.	n.d.	n.d.	fresh
2-Octen-1-ol	n.d.	n.d.	n.d.	n.d.	n.d.	n.d.	n.d.	n.d.	0.26 ± 0.08 ^a^	n.d.	0.92 ± 0.05 ^a^	n.d.	citrus, green
3-Buten-1-ol, 3-methyl-	n.d.	n.d.	n.d.	n.d.	n.d.	n.d.	n.d.	n.d.	n.d.	6.31 ± 0.10 ^b^	4.62 ± 0.04 ^a^	n.d.	sweet
1-Butanol, 3-methyl-	n.d.	n.d.	n.d.	n.d.	n.d.	n.d.	n.d.	n.d.	n.d.	6.31 ± 0.06 ^b^	5.05 ± 0.09 ^a^	n.d.	pear, pungent
2-Hexanol, 5-methyl-	n.d.	n.d.	n.d.	n.d.	n.d.	n.d.	n.d.	n.d.	n.d.	n.d.	0.99 ± 0.07 ^a^	0.30 ± 0.05 ^a^	fruits
**Aldehydes**													
Hexanal	40.37 ± 0.13 ^c^	46.09 ± 0.12 ^d^	18.23 ± 0.09 ^b^	13.19 ± 0.14 ^a^	n.d.	29.78 ± 0.12 ^b^	31.49 ± 0.15 ^c^	4.38 ± 0.11 ^a^	n.d.	4.19 ± 0.09 ^b^	n.d.	2.66 ± 0.08 ^a^	grass, fatty
Heptanal	5.07 ± 0.07 ^b^	2.69 ± 0.14 ^a^	n.d.	n.d.	0.65 ± 0.09 ^a^	2.07 ± 0.13 ^b^	n.d.	n.d.	n.d.	n.d.	n.d.	n.d.	fruits
Benzaldehyde	6.72 ± 0.10 ^c^	1.79 ± 0.06 ^ab^	1.13 ± 0.07 ^a^	0.71 ± 0.09 ^a^	1.13 ± 0.09 ^ab^	1.86 ± 0.09 ^b^	0.50 ± 0.11 ^a^	0.64 ± 0.11 ^a^	1.17 ± 0.05 ^ab^	2.80 ± 0.06 ^c^	0.85 ± 0.06 ^a^	0.30 ± 0.05 ^a^	almonds
Octanal	n.d.	1.15 ± 0.05	n.d.	n.d.	n.d.	0.45 ± 0.10	n.d.	n.d.	n.d.	n.d.	n.d.	n.d.	fatty, citrus
2-Heptenal, (Z)-	n.d.	n.d.	n.d.	n.d.	n.d.	0.91 ± 0.04 ^a^	0.71 ± 0.09 ^a^	n.d.	0.33 ± 0.07	n.d.	n.d.	n.d.	green, fatty
Benzeneacetaldehyde	n.d.	n.d.	n.d.	n.d.	n.d.	1.41 ± 0.11 ^ab^	0.87 ± 0.09 ^a^	n.d.	0.88 ± 0.10 ^a^	0.64 ± 0.09 ^a^	2.86 ± 0.08 ^b^	n.d.	floral
2-Octenal, (E)-	n.d.	n.d.	n.d.	n.d.	n.d.	0.65 ± 0.08 ^a^	0.97 ± 0.12 ^a^	n.d.	n.d.	n.d.	n.d.	n.d.	honey, nuts
Nonanal	n.d.	1.08 ± 0.09	n.d.	n.d.	n.d.	0.35 ± 0.10	n.d.	n.d.	n.d.	n.d.	n.d.	n.d.	fatty, citrus
Decanal	n.d.	0.58 ± 0.06	n.d.	n.d.	n.d.	n.d.	n.d.	n.d.	n.d.	n.d.	n.d.	n.d.	orange peel
**Ketones**													
Acetophenone	7.53 ± 0.10 ^c^	1.63 ± 0.09 ^ab^	2.55 ± 0.05 ^b^	0.81 ± 0.09 ^a^	1.01 ± 0.10 ^ab^	0.49 ± 0.07 ^a^	0.32 ± 0.12 ^a^	0.18 ± 0.03 ^a^	0.74 ± 0.11 ^a^	1.07 ± 0.03 ^ab^	0.63 ± 0.050 ^a^	0.29 ± 0.03 ^a^	floral
2-Heptanone	n.d.	0.58 ± 0.05 ^a^	1.79 ± 0.05 ^b^	1.89 ± 0.14 ^b^	0.30 ± 0.08 ^a^	2.12 ± 0.10 ^b^	1.73 ± 0.06 ^b^	0.53 ± 0.10 ^a^	n.d.	10.4 ± 0.04 ^b^	2.53 ± 0.06 ^a^	n.d.	banana
3-Octen-2-one	n.d.	n.d.	n.d.	0.11 ± 0.02	n.d.	n.d.	n.d.	n.d.	n.d.	n.d.	n.d.	n.d.	cranberries
2-Butanone, 3-hydroxy-	n.d.	n.d.	n.d.	n.d.	1.24 ± 0.05 ^a^	n.d.	n.d.	4.83 ± 0.10 ^b^	n.d.	1.60 ± 0.04	n.d.	n.d.	creamy
2-Nonanone	n.d.	n.d.	n.d.	n.d.	n.d.	n.d.	0.26 ± 0.09	n.d.	n.d.	2.14 ± 0.04 ^b^	0.93 ± 0.09 ^a^	n.d.	grass, fresh
**Acids**													
Butanoic acid	n.d.	n.d.	0.93 ± 0.08	n.d.	n.d.	n.d.	n.d.	n.d.	n.d.	0.86 ± 0.08	n.d.	n.d.	rancid cheese
Hexanoic acid	n.d.	n.d.	n.d.	n.d.	n.d.	14.99 ± 0.13	n.d.	n.d.	n.d.	n.d.	n.d.	n.d.	urea
Benzoic acid	n.d.	n.d.	n.d.	n.d.	n.d.	n.d.	n.d.	n.d.	0.64 ± 0.10 ^a^	n.d.	0.91 ± 0.07 ^a^	n.d.	balsamic
**Others**													
Butanoic acid, ethyl ester	n.d.	n.d.	n.d.	n.d.	55.99 ± 0.13 ^b^	n.d.	11.31 ± 0.09 ^a^	n.d.	57.34 ± 0.12 ^c^	n.d.	22.24 ± 0.06 ^b^	1.32 ± 0.04 ^a^	pineapple
Butanoic acid, propyl ester	n.d.	n.d.	n.d.	n.d.	3.39 ± 0.12	n.d.	n.d.	n.d.	0.72 ± 0.08	n.d.	n.d.	n.d.	apricots
Butanoic acid, butyl ester	n.d.	n.d.	n.d.	n.d.	0.45 ± 0.12	n.d.	n.d.	n.d.	0.37 ± 0.07	n.d.	n.d.	n.d.	pineapple
Hexanoic acid, ethyl ester	n.d.	n.d.	n.d.	n.d.	n.d.	0.83 ± 0.07 ^a^	n.d.	0.46 ± 0.11 ^a^	0.86 ± 0.07 ^a^	n.d.	n.d.	1.22 ± 0.05 ^ab^	fruits, sweet
Acetic acid, hexyl ester	n.d.	n.d.	n.d.	n.d.	n.d.	n.d.	0.30 ± 0.06	n.d.	n.d.	n.d.	n.d.	1.74 ± 0.10	sweet, green
1-Butanol, 3-methyl-, acetate	n.d.	n.d.	n.d.	n.d.	n.d.	n.d.	n.d.	n.d.	0.38 ± 0.06	n.d.	n.d.	n.d.	pear, banana
Acetic acid, pentyl ester	n.d.	n.d.	n.d.	n.d.	n.d.	n.d.	n.d.	n.d.	n.d.	n.d.	n.d.	0.23 ± 0.03	fruits, banana
Furan, 2-pentyl-	n.d.	2.35 ± 0.12 ^a^	2.42 ± 0.11 ^a^	3.14 ± 0.07 ^ab^	1.15 ± 0.05 ^a^	3.40 ± 0.10 ^b^	5.21 ± 0.09 ^c^	2.87 ± 0.08 ^b^	1.88 ± 0.08 ^a^	4.43 ± 0.04 ^bc^	4.66 ± 0.10 ^c^	3.59 ± 0.05 ^b^	earth, green
Limonene	n.d.	n.d.	2.39 ± 0.07	n.d.	n.d.	1.59 ± 0.07 ^b^	n.d.	0.66 ± 0.11 ^a^	n.d.	2.05 ± 0.08 ^b^	n.d.	0.85 ± 0.09 ^a^	citrus
D-Limonene	n.d.	n.d.	n.d.	1.30 ± 0.09	n.d.	n.d.	1.34 ± 0.08	n.d.	n.d.	n.d.	0.96 ± 0.08	n.d.	mint, citrus
Disulfide, dimethyl	n.d.	n.d.	n.d.	n.d.	4.72 ± 0.09	n.d.	n.d.	n.d.	7.41 ± 0.12	n.d.	n.d.	n.d.	garlic
Dimethyl trisulfide	n.d.	n.d.	n.d.	n.d.	n.d.	n.d.	n.d.	n.d.	1.24 ± 0.06	n.d.	n.d.	n.d.	sulfur
n.i.	n.d.	2.44 ± 0.17	n.d.	n.d.	n.d.	n.d.	n.d.	n.d.	n.d.	n.d.	n.d.	n.d.	-

where: n.d.—not detected; n.i.—not identified. Results are represented as mean values ± standard deviation (SD), mg/kg, *n* = 3. Different small letters show the significant difference (*p* < 0.05) between M_0_ (wheat flour sourdough), M_1_ (einkorn flour sourdough), M_2_ (spelt flour sourdough), and M_3_ (emmer flour sourdough) at the same moment (0, 12, 2 h). The references for “Odor perceptions” was: http://www.thegoodscentscompany.com/ (accessed on 20 January 2023) [136], http://www.flavornet.org/flavornet.html (accessed on 20 January 2023) [137].

## Data Availability

All related data and methods are presented in this paper. Additional inquiries should be addressed to the corresponding author.
